# Application of Entropy Method to Quantify Future Ecological Flow in the Yellow River Basin

**DOI:** 10.3390/e24010072

**Published:** 2021-12-31

**Authors:** Xinru Wang, Huijuan Cui

**Affiliations:** 1 Key Laboratory of Land Surface Patterns and Simulation, Institute of Geographic Sciences and Natural Resources Research, Chinese Academy of Sciences, Beijing 100101, China; wangxinru@igsnrr.ac.cn; 2 University of Chinese Academy of Sciences, Beijing 100049, China

**Keywords:** ecological flow, Yellow River basin, climate change, entropy

## Abstract

Due to both anthropogenic and climate change impacts, precipitation and runoff in the Yellow River basin have decreased in the past 50 years, leading to more pressure in sustaining human beings and ecosystem needs. It is essential to evaluate the flow condition in the Yellow River basin and see whether it may satisfy its ecological flow in the future. Therefore, this study applied an entropy-based method to calculate the flow duration curves from both observed and simulated data to evaluate the impact of climate change on ecological flow in the Yellow River basin. The simulated FDCs from H08 and DBH models show good agreement with each other and fit observation well. Results show that the decadal FDC at each station is generally predicted to be higher or stay in the higher range under both RCP 2.6 and 8.5 scenarios, suggesting an increase in water amount in the future. It is found that the high flows increase much faster than the low flows, resulting in larger slopes than the references ones, which is due to the larger entropy and M values in the future. At most of the stations, the future values of Q95 and Q90 will safely exceed the threshold. It is found that at the Lanzhou, Wubao, Longmen, and Huayuankou stations, there will be no or little threat to future ecological flow. Still, at the Toudaoguai and Sanmanxia stations, the ecological requirement is not always satisfied. The water stress at the Tangnaihai station from the upper stream of the Yellow River may be threatened in the future.

## 1. Introduction

The Yellow River, the second-largest river in China, is the cradle of Chinese civilization and plays an essential role in regional development. Still, the Yellow River basin (YRB) is highly populated nowadays, with about 110 million people, accounting for 9% of China’s total population [1]. However, the YRB has long been suffering from a water shortage for decades [2,3], as its annual average flow only accounts for 2% of China’s water resources. There were always competitions between human water needs and environmental flow due to the rapid economic growth over the basin [4]. During the last 50 years, due to anthropogenic and climate change impact, precipitation in the basin has shown a nonsignificant decrease, and runoff has significantly decreased [5], with greater variability in streamflow [6,7,8], leading to more pressure in sustaining human life beings and ecosystem needs. It is well recognized that the river discharge is one of the important drivers for the river ecosystems [9]. There is a minimum requirement of the flow rate to sustain living habits, which is often called the ecological flow or the environmental flow [10,11]. It usually includes the number, frequency, time, and duration of flow events to sustain freshwater, estuarine, and nearshore ecosystems along with the dependent human livelihoods [12,13,14]. It is necessary to carry out ecological flow assessment and the amount of water needed for ecosystem protection and resource protection [12].

Many ecologically relevant hydrological indicators can be used to quantify the hydrological alterations on the flow regimes [9,15]. Low flow, such as Q90 and Q95, is often used as an indicator of whether the flow is capable of maintaining the health of the river ecosystem [16]. According to U.S. EPA [17], the flow between 90% and 100% can be classified into low flow to facilitate diagnostic and analytical applications of flow. Q90 and Q95 can be determined from the flow duration curves (FDCs), a plot of streamflow values against the percentage of time that the flow equals or exceeds a specific value. The flow duration curve is usually drawn according to the percentage of time that the flow equals or exceeds the flow in a year, which can effectively reflect the runoff characteristics of the basin from high flow to low flow under various flow conditions [18]. FDC is constructed over a full range of time, scaled from 0% to 100%. If the FDC is based upon the long-term flow of a stream, then it can be employed for predicting the distribution of future flows for water supply [19], hydropower [20], sediment load [21], and pollution [22]. FDCs are often calculated empirically. Singh et al. [23] first introduced the entropy concept to compute FDCs, which shows an advantage in estimating parameters and forecasting. Besides, the entropic parameter is capable of representing the slope of the FDC, which is an indicator of the variability of the flow [23,24].

Although many studies have addressed the ecological impact of the Yellow River hydrological changes [25,26,27] and have shown different degrees of ecological risks from upper to lower basin [28], it is not clear whether water stress will further intensify the ecological conditions under changing climate [29]. Therefore, this paper aimed to employ the entropy-based method to analyze the changes in low flows, especially Q90 and Q95 from the FDCs. To that end, FDCs are calculated from both observed and simulated streamflow from two global hydrological models under two climate change scenarios.

## 2. Methods

Using the entropy method, the FDC is estimated from the monthly streamflow, both observed and simulated. It should be noted that FDCs are usually calculated based on daily discharge, but while considering the long period from 1961 to 2100, decadal averaged discharge is used to calculate FDCs.

### 2.1. Entropy Theory

Shannon [30] developed entropy as a measure of information or uncertainty. Later, Jaynes [31] developed the principle of maximum entropy, which states that the best estimate of current states of knowledge (best fit of the probability distribution) is the one with the maximum entropy. Such properties allow wide application in hydrological and hydraulic engineering, for example, estimation of velocity distribution [32,33,34], probability distribution [35], and flow forecasting [36]. The Shannon entropy and the Tsallis entropy [37] have been applied to estimate the flow duration curves and have shown an advantage in resolution compared to the traditional method [38]. In this study, the Shannon entropy is used to estimate the flow duration curves of YRB. The main steps are as follows.

Assuming that temporally averaged streamflow *Q* is a random variable, varying from the minimum value *Q*_min_ to the maximum value *Q*_max_, with the probability distribution *f*(*Q*), the Shannon entropy of the streamflow can be expressed as

(1) $$H=-\int_{Q_{\min}}^{Q_{\max}}f(Q)ln[f(Q)]dQ$$

Equation (1) measures the uncertainty in determining *f*(*Q*). Then the principle of maximum entropy is applied with a constraint to the mean runoff, resulting in the least-biased distribution of the streamflow as

(2) $$f(Q)=\exp[-\lambda_{0}-\lambda_{1}Q]$$

where *λ*_0_ and *λ*_1_ are the Lagrange multipliers used to maximize the entropy.

### 2.2. Entropy-Based Flow Duration Curve

The next step is to relate the flow duration curve with the entropy-based probability distribution, and it is necessary to assume the relationship between the cumulative probability distribution function (CDF) of *Q* and time. According to Singh et al. [31], since the flow duration curve is a relation between flow discharge and the percentage of the time the flow is equaled or exceeded, the cumulative probability distribution *F*(*Q*) = *P* (discharge ≤ a given value of *Q*), *P* = probability, can be expressed as

(3) $$F\left(Q\right)=1-a{\left(\frac{t}{T}\right)}^{b}$$

where *a* and *b* are coefficients that can be estimated by empirical fitting, *t* is the time (number of months in this study) the flow equals or exceeds, and *T* is the total number of months. Differentiating Equation (3) with respect to *Q*, one obtains

(4) $$dF(Q)=f(Q)dQ=-ab{\left(\frac{1}{T}\right)}^{b}t^{b-1}dt$$

Then, substituting Equation (2) into Equation (4) and integrating Equation (4) from *Q*_min_ to the maximum value *Q*_max_, one obtains

(5) $$Q=-\frac{1}{\lambda_{1}}\ln\left\{\exp(-\lambda_{1}Q_{\max})-[\exp(-\lambda_{1}Q_{\max})-\exp(-\lambda_{1}Q_{\min})]a{\left(\frac{t}{T}\right)}^{b}\right\}$$

Let us define the entropy parameter $M=\lambda_{1}Q_{\max}$ and assume that $Q_{\min}≅0$; then, the FDC can be finally written as follows:

(6) $$\frac{Q}{Q_{\max}}=-\frac{1}{M}\ln\left\{\exp(-M)-[\exp(-M)-1]a{\left(\frac{t}{T}\right)}^{b}\right\}$$

Equation (6) expresses the relationship between the streamflow and time, which can be used to determine the FDC. It can be seen that Equation (6) contains only parameter *M*, which can be seen as an index of the uniformity of the probability distribution of streamflow and is related to the ratio between *Q*_mean_ and *Q*_max_ [31]. Therefore, once a series of time-averaged *Q* is observed, one can calculate *Q*_mean_ and *Q*_max_ from the observation and then calculate the entropy parameter *M*. The FDC curves can be plotted by inputting *M* into Equation (6) for exceedance probabilities from 0 to 1.

## 3. Data

**Figure 1:**
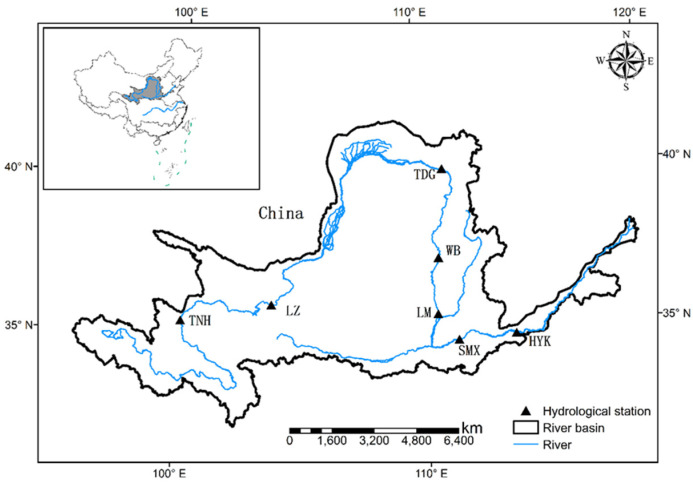
Map of study area with selected hydrological stations.

As shown in Figure 1, the Yellow River originates from the inland of Northwest China and flows into the Bohai Sea, and occupies a drainage area of 795,000 square kilometers. Most area of the basin belongs to the arid and semiarid region dominated by a continental monsoon climate, with the annual temperature ranging from 4 °C to 14 °C, and the annual mean precipitation varying from 360 mm to 670 mm (Yang et al. 2010). The observed monthly streamflow data at seven selected hydrological stations (Figure 1) from upstream to downstream covering the whole basin, during 1961 and 2012, are obtained from the *Hydrological Statistics Year Books*, and their basic information is summarized in Table 1. The annual mean streamflow generally increases from the upstream to downstream stations, while Lanzhou is an exception.

To examine the impact of climate change, simulated monthly runoff data from 1971 to 2099 ran by two GHMs, the distributed biosphere hydrological (DBH) model and the H08 model, are used. These two models are selected because both of their simulations have been verified in the Yellow River basin [39]. The simulated monthly runoff data are generated with the climate forcing of five global climate models (GCMs), namely, HadGEM2-ES, IPSL-CM5A-LR, MIROC-ESM-CHEM, GFDL-ESM2M, and NorESM1, under two representative concentration pathways, RCP 2.6 and RCP 8.5. Due to the mismatch between gridded simulated data and observed stational data in terms of resolution and scale, the bias correction is applied to the simulated data as described by Piani et al. [40]:

(7) $$R_{BC}(t)=\bar{R_{O,REF}}+\frac{\sigma_{O,REF}}{\sigma_{T,REF}}(R_{RAW}(t)-\bar{R_{M,REF}})$$

where $R_{BC}(t)$ is the bias-corrected simulated runoff, $R_{RAW}(t)$ is a raw simulated runoff, $\bar{R_{O,REF}}$ and $\bar{R_{M,REF}}$ are the mean of the observed and simulated runoff in the reference period (1971–2009), and $\sigma_{T,REF}$ and $\sigma_{O,REF}$ represent the standard deviation of the simulated and observed runoff in the reference period, respectively.

## 4. Results

### 4.1. Validation of FDC with the Observed Data

**Figure 2:**
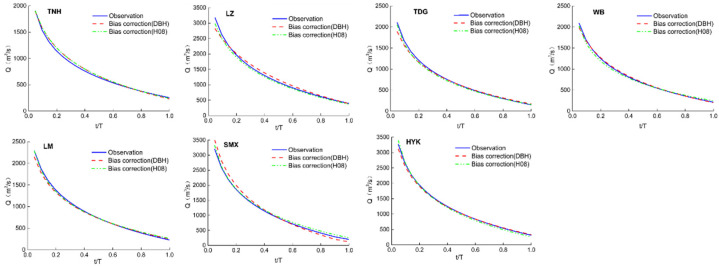
Verification of flow duration curves from simulations of DBH and H08 at seven hydrological stations: Tangnaihai (TNH), Lanzhou (LZ), Toudaoguai (TDG), Wubao (WB), Longmen (LM), Sanmenxia (SMX), and Huayuankou (HYK).

After bias correction, the FDC calculated from both DBH and H08 is compared to the FDC using the series observed from 1971 to 2009 in Figure 2. At most stations, the simulated FDCs fit observation well with R^2^ up to 0.98 (Table 2), and little deviation is found in *Q*_max_ and *Q*_min_. Specifically, for the Lanzhou and Toudaoguai stations, the simulated FDCs have lower estimation than observation for frequencies less than 0.2, showing a divergence less than 10% from the observation. Compared to the result of H08, the DBH model simulates about 6% higher than the observed value for the middle frequencies between 0.2 and 0.8, indicating that the simulated FDCs tend to be flatter than those observed. This is more visible from the Sanmenxia station, where the simulated FDC by DBH is slightly higher for low frequencies and slightly lower for high frequencies. In general, the two simulated models show good agreement with each other at all stations, and therefore, the average value of the two models are used hereafter.

### 4.2. Prediction of Future FDCs

**Figure 3:**
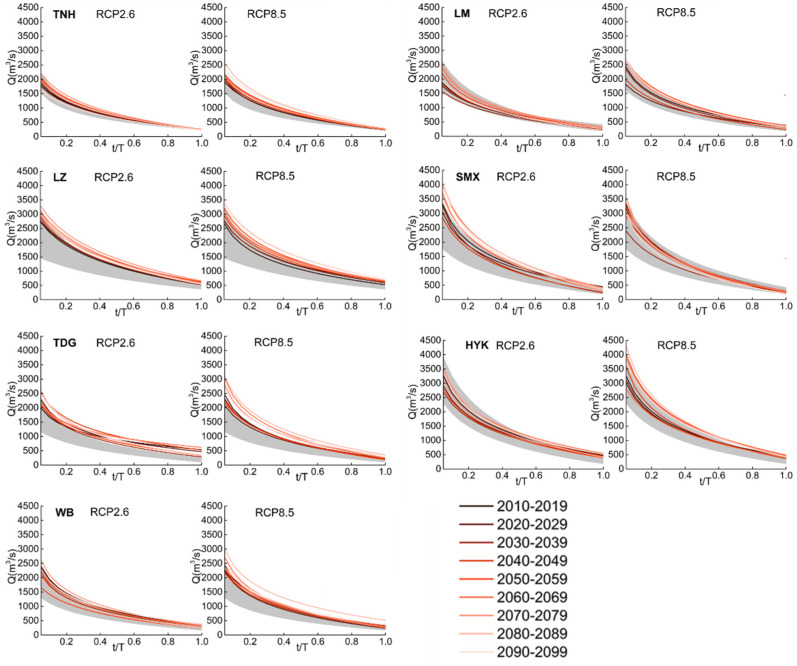
Predicted decadal FDCs under RCP 2.6 and 8.5 for 2010–2099 with reference range obtained from 1971 to 2009 (shaded area) for each station in the YRB.

Compared to the historical range during 1971–2009, the decadal FDC at each station is generally higher or stays in the higher range under both RCP 2.6 and 8.5 scenarios (Figure 3), suggesting a future increase in the volume of water. Compared to other stations, the Tangnaihai station from the upper stream of the Yellow River shows the least increase. Although FDCs increase over time in general, they do not increase monotonically. The highest FDCs will mostly occur during the 2070s or 2080s and then decrease after that. It is noted that the increase rates in high flows and low flows are different. At most stations, the high flows increase much faster than the low flows; as a result, the predicted FDCs have larger slopes than the references ones. *Q*_max_ at all stations increases by 70–750 m^3^/s, while *Q*_min_ only increases by 30–200 m^3^/s at all stations. This implies that higher flows will be more impacted by climate change than lower flows. Besides, the predicted FDCs under the RCP 8.5 scenario are generally higher than those under RCP 2.6, which is about 3–24% higher. However, the Sanmenxia station is an exception, where *Q*_max_ varies from 2700 to 4000 m^3^/s under RCP 2.6, which will be 2400–3500 m^3^/s under RCP 8.5. The FDCs under the RCP 8.5 scenario are more concentrated than those under RCP 2.6, which is also unusual in comparison to other stations.

**Figure 4:**
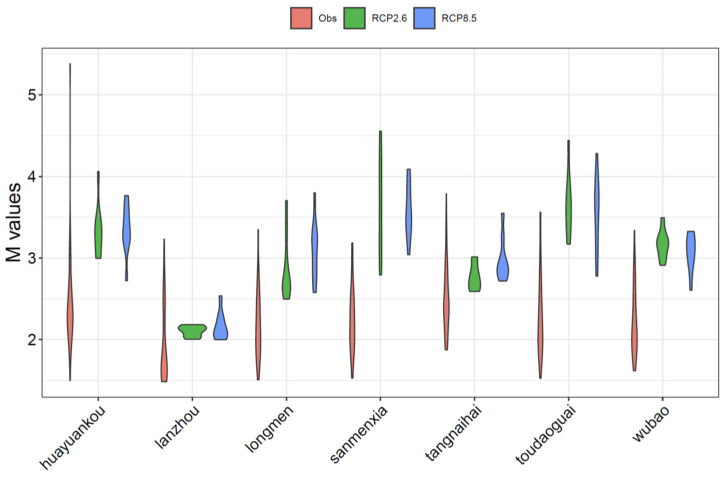
Change in the *M* value over the years.

As suggested by Singh et al. (2014) and Zhang et al. (2017) [23,24], the entropy parameter *M* represents the uniformness of the distribution, and a higher *M* value implying higher entropy leads to a more uniform distribution from high to low flows. Figure 4 plots the *M* values of decadal FDCs compared to the historical period. In general, the *M* values get larger in the future, while the variations become smaller, compared to the values in 1971–2009. This implies that the FDC will become more uniform with increased values of *Q*_max_, which is consistent with the previous results (Figure 3). Besides, the *M* values under RCP 2.6 and RCP 8.5 do not have significant differences in mean values, but the *M* values under RCP 2.6 show smaller variation compared to those under RCP 8.5 at most stations, except for Huayuankou and Sanmenxia. This indicates that the FDCs under RCP 2.6 are more consistent than those under RCP 8.5.

**Figure 5:**
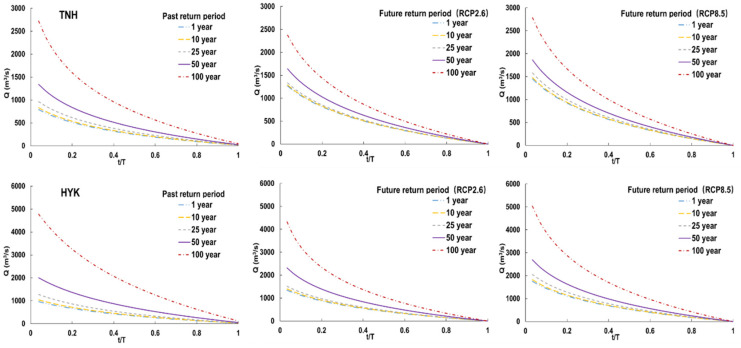
The predicted FDCs of different return periods at the Tangnaihai and Huayuankou stations.

Then, the FDCs of different return periods are calculated with the corresponding *Q*_max_ of different return periods and compared to the historical observation (Figure 5). For different return periods from 1 year to 50 years, most FDCs increase by 2–83%, which is larger under RCP 8.5 than RCP 2.6. As the return period extends to 100 years, the increase in the FDC gets slower or stops. The FDC of the 100-year return period is seen to be lower than the historical observation at both stations, with a 2–12% decrease at the Tangnaihai station and 5–9% decrease at the Huayuankou station, which is less under RCP 8.5 than RCP 2.6. This leads to fewer differences between the FDCs of different return periods.

### 4.3. Predicted Ecological Flow

**Figure 6:**
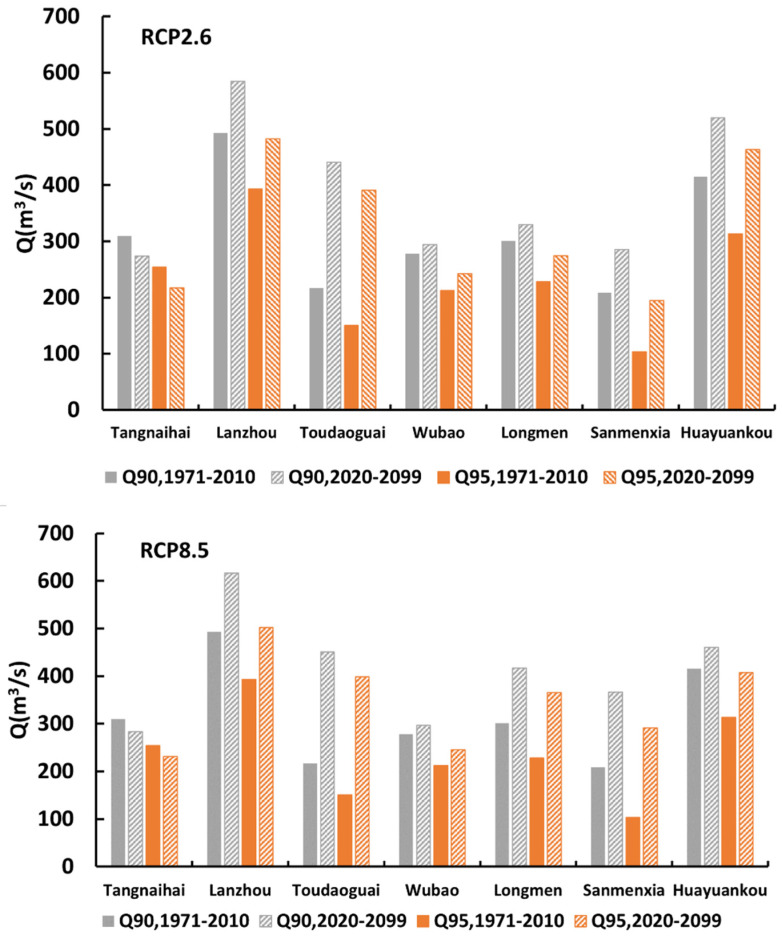
The changes in the ecological flow values of each hydrological station.

Finally, the change in Q95 and Q90 from the FDC is examined to see the impact of climate change on ecological flow (Figure 6). The Q95 and Q90 during 1971–2009 can be regarded as extremely low ecological flow and low ecological flow, respectively, and be considered as the threshold to examine whether the predicted future flow will sustain the ecology and environment. As seen in Figure 6, the future values of Q95 and Q90 at most stations will safely exceed the threshold, with the exception of the Tangnaihai station under RCP 2.6 and the Sanmenxia station under RCP 8.5. This indicates that, in general, the ecological flow will meet the least requirement in the future. Specifically, under RCP 2.6, Q90 and Q95 increase the least at the Longmen station, from 307 to 351 m^3^/s and from 227 to 293 m^3^/s, respectively, while at the Toudaoguai station, Q90 and Q95 increase the most, from 216 to 440 m^3^/s and 150 to 390 m^3^/s, respectively. The future ecological flow values of Tangnaihai, Toudaoguai, Wubao, Longmen, and Sanmenxia show little difference; Q90 is within 100 m^3^/s and Q95 is within 136 m^3^/s. Besides, at all stations, Q90 and Q95 under RCP 8.5 are larger than those under the RCP 2.6 scenario, implying that the ecological requirements are met to a greater extent.

**Figure 7:**
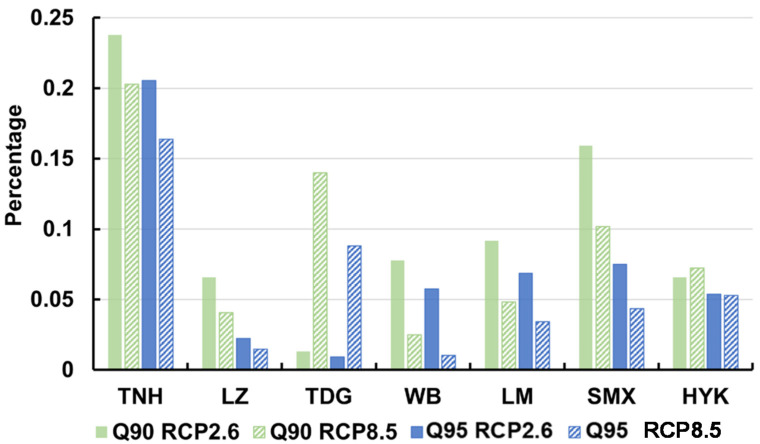
The percentage of the ecological flow value from 2010 to 2099 that is lower than the total runoff value of the ecological flow in the previous period.

We also calculated the number of days when the flow will less than the Q90 and Q95 values in the future and examined the percentage of days lower than the threshold in Figure 7. If Q90 is greater than 10% and Q95 is greater than 5%, there is a certain risk of ecological flow. As the predicted Q90 and Q95 values exceed the historical threshold in Figure 6, the number of days with flow lower than the threshold decreases in the future at most stations. It is found that at the Lanzhou, Wubao, Longmen, and Huayuankou stations, there is no or little threat to future ecological flow since the number of days with flow lower than Q90 and Q95 are less than 10% and 5%. However, at the Toudaoguai and Sanmanxia stations, the ecological requirement is not always satisfied. There is a risk at the Toudaoguai station under the RCP 8.5 scenario, and at the Sanmenxia station under the RCP 2.6 scenario. However, the Tangnaihai station will be mostly dangerous in the future, with the number of days with flow lower than Q90 exceeding 20% and the number of days with flow lower than Q95 exceeding 15%, which is 2–3 times higher than the requirement. This implies that water stress may be further exacerbated in the upstream area.

## 5. Discussion

The derivation of the FDC by the entropy method is straightforward, and the only assumption made is about the relationship between the cumulative probability distribution function (CDF) of *Q* and time. In this study, we followed Cui and Singh [41], assumed a nonlinear relationship between the CDF of *Q* and time, and calculated the exponentials of CDF, *a* and *b* in Equation (3), using the least-squares method. This ensures the general applicability of the method and that it can be applied to any station in the YRB, not just the seven used in this study.

The entropy parameter *M*, which can represent the uniformness of the distribution, is another major advantage of using the entropy method. If the FDC of one cross section is consistent over time, the parameter *M* would not change. However, as this study shows, the FDCs at all stations, as well as the entropy parameter *M*, change over time. It may be due to both climate change and anthropogenic activities influencing the rainfall and runoff process, which leads to the nonstationary problem. Unfortunately, the proposed method has not yet considered nonstationary issues, which should be further studied in the future.

## 6. Conclusions

This study applied the entropy-based method to calculate the flow duration curves to evaluate the impact of climate change on ecological flow for the Yellow River basin. The results show that climate change will have a certain impact on ecological flow in the Yellow River basin, especially in the upper reaches of the Yellow River. The following conclusions can be drawn.

1. The simulated FDCs from the H08 and DBH models show good agreement with each other and fit observation well. The decadal FDC at each station is generally predicted to be higher or to stay in the higher range under both RCP 2.6 and 8.5 scenarios, implying a future increase in the volume of water.
2. It is noted that the rates of increase in high flows and low flows are different, and at most stations, high flows increase much faster than low flows. As a result, the predicted FDCs have larger slopes than the references due to the larger *M* values in the future.
3. At most of the stations, the future values of Q95 and Q90 will safely exceed the threshold. However, the number of days with flow lower than the threshold may be more than 5% and 10%, indicating a certain risk of ecological flow. It is found that there will be no or little threat to future ecological flow at the Lanzhou, Wubao, Longmen, and Huayuankou stations, but the ecological requirement is not always satisfied at the Toudaoguai and Sanmanxia stations.
4. Water stress at the Tangnaihai station from the upper stream of the Yellow River may be threatened in the future. The FDCs of the Tangnaihai station show the least increase, with the number of days with flow lower than Q90 exceeding 20% and the number of days with flow lower than Q95 exceeding 15%, which is 2–3 times higher than the requirement. This indicates that water stress may be further exacerbated in the upstream area.

## Figures and Tables

**Table 1:** Statistics of streamflow data at the seven selected hydrological stations.

Stations	Location	Record Length	Annual Mean Streamflow (m^3^/s)	Lowest Value (m^3^/s)
Tangnaihai (TNH)	Upstream	1961–2012	638.81	87.10
Lanzhou (LZ)	Upstream	1967–2012	941.48	285.58
Toudaoguai (TDG)	Upstream	1961–2012	618.41	65.55
Wubao (WB)	Midstream	1961–2012	667.42	68.68
Longmen (LM)	Midstream	1961–2012	732.50	120.17
Sanmenxia (SMX)	Midstream	1961–2012 (missing 2006)	888.06	111.41
Huayuankou (HYK)	Downstream	1961–2012	1008.37	126.88

**Table 2:** Comparison statistics of simulated and observed FDCs.

Station	Q_max_ (m^3^/s)			Q_min_ (m^3^/s)			R^2^ *	
	DBH	H08	Obs.	DBH	H08	Obs.	DBH	H08
TNH	1890.49	1900.57	1904.07	231.22	240.16	254.07	0.899	0.895
LZ	2507.88	2504.04	2630.16	385.82	367.01	393.04	0.956	0.929
TDG	1887.16	2043.32	2102.30	170.11	155.06	150.27	0.896	0.864
WB	1980.74	2022.15	2086.77	200.51	245.91	212.53	0.908	0.865
LM	2144.30	2281.19	2287.47	251.14	262.73	227.78	0.889	0.868
SMX	3499.19	3320.21	3197.39	117.85	251.04	199.76	0.881	0.864
HYK	3116.28	3391.48	3268.97	331.53	262.17	313.10	0.885	0.866

* R^2^ represents the correlation between the simulated and observed FDCs.

## Data Availability

The data presented in this study are available on request from the corresponding author.
